# Peroral Cholangioscopy-Guided Forceps Biopsy and Endoscopic Scraper for the Diagnosis of Indeterminate Extrahepatic Biliary Stricture

**DOI:** 10.3390/jcm8060873

**Published:** 2019-06-19

**Authors:** Masayuki Kato, Takumi Onoyama, Yohei Takeda, Soichiro Kawata, Hiroki Kurumi, Hiroki Koda, Taro Yamashita, Wataru Hamamoto, Yuri Sakamoto, Kazuya Matsumoto, Hajime Isomoto

**Affiliations:** Division of Medicine and Clinical Science, Department of Multidisciplinary Internal Medicine, Faculty of Medicine, Tottori University, Nishi-cho 36-1, Yonago 683-8504, Japan; m06027mk@jichi.ac.jp (M.K.); yhytkd7@outlook.jp (Y.T.); kawataso0527@yahoo.co.jp (S.K.); kurumi_1022_1107@yahoo.co.jp (H.Ku.); hkoda@aichi-cc.jp (H.Ko.); yamat11@gmail.com (T.Y.); hamamoto_trr@yahoo.co.jp (W.H.); yuri.sakamoto@mac.com (Y.S.); matsumotokazuya@tottori-u.ac.jp (K.M.); isomoto@med.tottori-u.ac.jp (H.I.)

**Keywords:** peroral cholangioscopy, trefle, cholangiocarcinoma, accuracy, adverse event

## Abstract

Background: Peroral cholangioscopy (POCS) has become a widely-used technique in diagnosing indeterminate biliary strictures, enabling optical viewing of the biliary system and targeted biopsies under direct vision. The diagnostic utility of the new endoscopic scraper, Trefle^®^, for extrahepatic cholangiocarcinoma (ECC) has also been reported. However, the diagnostic utility of POCS-guided and Trefle^®^-assisted tissue acquisition for ECC has never been compared empirically. We evaluated the efficacy and safety of Trefle^®^-assisted tissue acquisition for diagnosing ECC compared with POCS-guided tissue sampling. Methods: Patients who underwent Trefle^®^-assisted tissue acquisition or POCS-guided forceps biopsy to differentiate ECC from benign biliary disease between April 2014 and March 2018 were enrolled retrospectively. We evaluated the diagnostic performance of Trefle^®^-assisted tissue acquisition and POCS-guided forceps biopsy based on pathological evaluation. We also compared adverse events associated with Trefle^®^-assisted tissue acquisition with those of POCS-guided forceps biopsy. Results: We enrolled 34 patients with biliary disease and performed Trefle^®^-assisted tissue acquisition and POCS-guided forceps biopsy in 14 and 20 patients, respectively. Sensitivity, specificity, and accuracy of Trefle^®^-assisted tissue acquisition were 87.5%, 83.3%, and 85.7%, respectively, and for POCS-guided forceps biopsy, these were 90.0% each. Statistical values of Trefle^®^-assisted tissue acquisition and POCS-guided tissue acquisition were not significantly different. There were no significant differences in the occurrence of adverse events between the Trefle^®^-assisted tissue acquisition and the POCS-guided forceps biopsy (35.7% vs. 25.0%, *p =* 0.770). Compared with patients who underwent POCS procedure, endoscopic sphincterotomy was performed for fewer patients who underwent Trefle^®^-assisted tissue acquisition (*p <* 0.001). Conclusions: The diagnostic ability of Trefle^®^-assisted tissue acquisition for ECC is similar to that of POCS-guided tissue acquisition. Trefle^®^-assisted tissue acquisition might also help to preserve the sphincter of Oddi and its digestive function.

## 1. Introduction

Extrahepatic cholangiocarcinoma (ECC) is a disease with a usually poor prognosis, with a five-year survival rate of 20.5% (median survival time, 11.3 months), because it is often diagnosed at an advanced stage and is often unresectable [1]. The prognosis of ECC might improve if the disease is diagnosed early [2]. However, it is often difficult to differentiate between ECC and benign biliary strictures, such as primary sclerosing cholangitis, immunoglobulin G subclass 4 (IgG4)-associated sclerosing cholangitis, and Mirizzi syndrome [3]. It is essential to distinguish ECC from benign biliary disease, as the treatment strategies and prognoses differ significantly.

Endoscopic retrograde cholangiopancreatography (ERCP) is a common method for tissue acquisition in patients with indeterminate biliary strictures, using bile aspiration cytology, biliary brush cytology, and forceps biopsy. The specificity of pathological examination of tissues obtained by ERCP for biliary strictures is nearly 100%. Obtaining histological or cytological evidence is very important to determine appropriate therapeutic strategies for ECC patients. However, the sensitivities of bile aspiration cytology, brush cytology, and forceps biopsy of biliary strictures are unsatisfactory, with a range of 6–72% [4,5]. 

In recent years, peroral cholangioscopy (POCS) has been commonly used to diagnose indeterminate biliary strictures. POCS-guided forceps biopsy was also a common method of tissue acquisition in biliary stricture. The sensitivity of POCS-guided forceps biopsy for ECC was higher than that of brush cytology, on the other hand, and was similar to that of fluoroscopy forceps biopsy [6,7]. Meanwhile, Sakuma et al. reported that a new endoscopic scraper, Trefle^®^ (PB7-3L5S, Piolax Medical Devices, Inc, Yokohama, Japan), had a higher cancer detectability than that of fluoroscopy forceps biopsy [8]. Trefle^®^ is a new device for sampling an adequate amount of tissue and bile juice in biliary stricture by ERCP. Although the sensitivity of POCS-guided forceps biopsy and Trefle^®^ reported is in the range of 60.1–64.7% [8,9], there have been no studies comparing the diagnostic utility of POCS-guided biopsy and that of Trefle^®^ for ECC. In this study, we examined the diagnostic performance and associated adverse events of Trefle^®^-assisted tissue acquisition and POCS-guided forceps biopsy for biliary strictures.

## 2. Materials and Methods

### 2.1. Study Population

In the study, we enrolled 34 patients with biliary disease retrospectively between April 2014 and March 2018 at our hospital. Inclusion criteria were as follows: (1) patients who underwent ERCP tissue acquisition with Trefle^®^ and/or POCS to differentiate cholangiocarcinoma from benign biliary disease; (2) patients aged 20 years or older when endoscopic procedures were performed. Exclusion criteria were as follows: (1) Patients who have not obtained consent; and (2) patients who have been receiving chemotherapy for malignant tumors within one month prior to the acquisition of pathological specimens.

Participants included 19 men and 15 women aged 47–85 years (median age, 71 years). Eighteen patients had ECC and 16 had benign lesions (Table 1). We measured tumor size in EUS or computed tomography (CT). Length of stricture were measured in ERCP. The Trefle^®^ group was defined as the patients who were performed Trefle^®^ as the first modality to tissue acquisition for biliary lesion, the POCS group was also defined in the same way. We evaluated the diagnostic ability of Trefle^®^ and that of POCS-guided tissue acquisition for ECC on the basis of the pathological evaluation. Furthermore, we compared adverse events in Trefle^®^ with those in POCS. This study was performed according to the guidelines described in the Helsinki Declaration for biomedical research involving human participants. The study was approved by the institutional review board of Tottori University (approval number: 18A205). Informed consent was obtained from all participants using an opt-out approach in this retrospective study.

### 2.2. Endoscopic Procedure

We performed ERCP using a side-viewing duodenoscope (JF260V/TJF240V; Olympus Optical Co., Ltd., Tokyo, Japan). We also used a 0.035-inch hydrophilic guidewire (M00556051; Boston Scientific Corporation, Natick, MA, USA) and/or a 0.025-inch hydrophilic guidewire (G-260-2545A; Olympus Optical Co., Ltd. MTA0025N48S; Medico’s Hirata, Inc, Osaka, Japan. M00556700; Boston Scientific Corporation) during ERCP.

**Figure 1:**
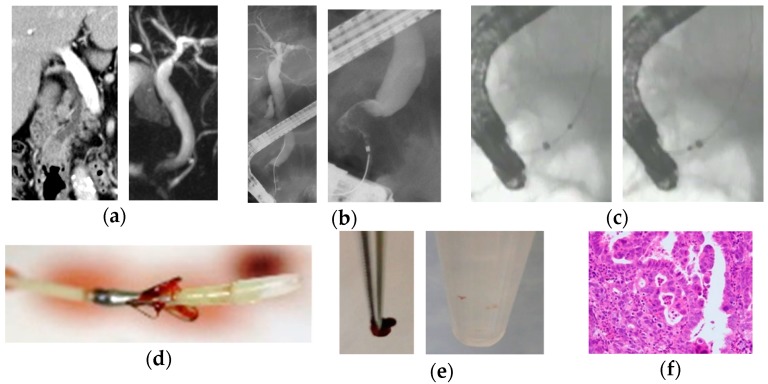
A case of bile duct stricture diagnosed as distal cholangiocarcinoma with an endoscopic scraper (Trefle^®^). (**a**) Computed tomography scan and magnetic resonance cholangiopancreatography showed stenosis in the distal bile duct; (**b**) endoscopic retrograde cholangiography showed irregular stenosis in the distal bile duct; (**c**) tissue acquisition procedure by Trefle^®^ was performed for the stenosis in the distal bile duct; (**d**) the appearance of Trefle^®^ showed. The inner sheath includes a 0.635-mm guidewire lumen and three metallic loops for scraping. Bile juice can be aspirated from the space between the inner and outer sheath; (**e**) tissue specimens and cell samples obtained by Trefle^®^; and (**f**) microscopic appearance of a hematoxylin and eosin-stained tissue sample appeared. The pathological diagnosis was adenocarcinoma.

The new endoscopic scraper, Trefle^®^ (PB7-3L5S; Piolax Medical Devices, Inc, Yokohama, Japan), having three metallic loops 1.6 mm in diameter and oriented at an angle of 120°, was inserted into the bile duct over the guidewire. Next, the loops of the device were opened and passed through the stricture under X-ray fluoroscopy. Then, scraped tissues and/or cell samples with bile juice were obtained by aspiration through the side port of the outer sheath into a 20 mL syringe (Figure 1).

**Figure 2:**
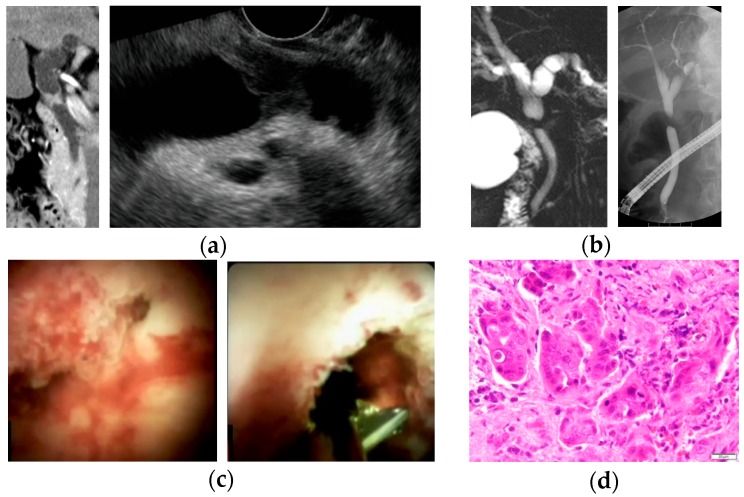
A case of bile duct stricture diagnosed as perihilar cholangiocarcinoma with peroral cholangioscopy (POCS)-guided forceps biopsy. (**a**) Computed tomography scan and endoscopic ultrasonography showed an irregular nodule in the perihilar bile duct; (**b**) magnetic resonance cholangiopancreatography and endoscopic retrograde cholangiography revealed irregular stenosis in the perihilar bile duct; (**c**) POCS showed the irregular papillary mucosa that existed from the bifurcation of the cystic duct to the perihilar bile duct. POCS-guided forceps biopsy was performed for the biliary stricture in the perihilar bile duct; and (**d**) hematoxylin and eosin staining revealed adenocarcinoma in specimens obtained from the biliary stricture.

POCS was performed using mini endoscopy (92176864-01A; Boston Scientific Corporation) direct visualization system. A cholangioscope was inserted into the bile duct over the guidewire, and POCS-guided forceps biopsy under direct vision was performed with M00546270 (Boston Scientific Corporation) with a 1.0-mm diameter cup. Bile was obtained by aspiration through the working channel lumen of the cholangioscope from the bile duct (Figure 2). 

Endoscopic sphincterotomy (EST) was carried out for difficult cases, whereby the endoscopic devices are inserted into the bile duct using a sphincterotome (KD-V411M-0725; Olympus Optical Co., Ltd.), if it was not previously performed and it was necessary.

### 2.3. Diagnostic Criteria

The diagnosis of cholangiocarcinoma was based on pathological diagnosis of bile aspiration cytology, transpapillary forceps biopsy, EUS-FNA, or surgical specimen. Cytodiagnosis of the specimens was performed using Papanicolaou’s method. Biopsy specimens were stained with hematoxylin and eosin and, if necessary, immunostaining, including Ki-67 and p53, was also performed. In histological findings, malignancy or suspected malignancy was considered positive. Patients with benign disease had a final diagnosis based on clinical and radiological follow-up data after 12 months or more.

### 2.4. Statistical Analysis

Statistical analysis was performed using StatFlex ver. 6.0 for Windows (Artech Co, Ltd., Osaka, Japan). Categorical variables were compared using the chi-square test. Continuous variables were compared by using the Mann–Whitney U-test. All values are expressed as means ± standard deviation or means with interquartile ranges. *p <* 0.05 was considered significant. 

## 3. Results

### 3.1. Patient’s Characteristics and Baseline Evaluation

**Figure 3:**
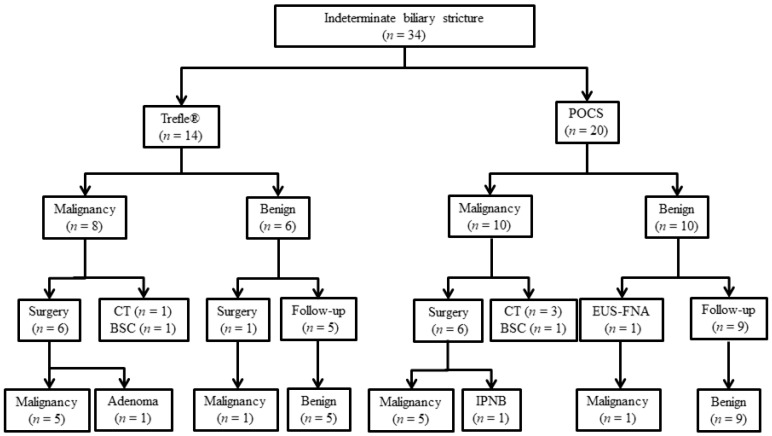
Diagnostic flowchart of patients included in the study. Abbreviations: POCS, peroral cholangioscopy; CT, chemotherapy; BSC, best supportive care; EUS-FNA, endoscopic ultrasonography-guided fine needle aspiration; IPNB, intraductal papillary neoplasm of the bile duct.

The characteristics of patients with biliary disease are shown in Table 1 and Table 2. In this study, the malignant group included six patients with perihilar cholangiocarcinoma and 12 patients with distal cholangiocarcinoma. Macroscopic types of ECC included two papillary-type, 12 nodular-type, and four flat-type. The benign group included 11 patients with benign biliary strictures, one with IgG4-associated sclerosing cholangitis, one with primary sclerosing cholangitis, one with a peribiliary cyst, one with intraductal papillary neoplasm of the bile duct, and one with bile duct adenoma. The final clinical diagnosis was derived from surgical pathology in 13 patients (Figure 3). We performed Trefle^®^-assisted tissue acquisition for 14 patients (Trefle^®^ group) and POCS-guided forceps biopsy for 20 patients (POCS group). All patients underwent bile aspiration cytology, except one patient who underwent POCS-guided forceps biopsy.

Table 2 summarizes the characteristics of all patients in the Trefle^®^ and POCS groups. There was no significant difference in age, sex, length of stricture, location of stricture, and presence of cholangitis between the two groups. There were no significant differences in the median level of serum total bilirubin (T-Bil), carcinoembryonic antigen (CEA), and carbohydrate antigen 19-9 (CA19-9) between the two groups.

The median number of biopsies was 2 (range, 1–6) and 3 (range, 2–9) in the Trefle^®^ group and the POCS group, respectively. The number of biopsies performed in the Trefle^®^ group was significantly fewer than that in POCS group (*p =* 0.0498). One patient in the Trefle^®^ group and five patients in the POCS group had previously undergone EST. EST was performed in all patients with naïve papilla who underwent the POCS procedure, except one patient on whom a precut papillotomy with a needle knife (9913023121; MTW Endoskopie W. Haag KG, Wesel, Germany) was performed. In the Trefle^®^ group, only three patients received EST. The patients who underwent EST or precut papillotomy in the Trefle^®^ group were significantly fewer than in the POCS group (*p <* 0.001). There was no significant difference between the median procedure time of Trefle^®^-assisted tissue acquisition and that of POCS-guided forceps biopsy (*p =* 0.766), lasting 92.0 minutes (range, 57–129) and 85.5 minutes (range, 34–170), respectively (Table 3).

### 3.2. Diagnostic Ability of Trefle^®^ and POCS-Guided Tissue Acquisition for Extrahepatic Cholangiocarcinoma

Table 4 summarizes the diagnostic performance of bile aspiration cytology, transpapillary biliary forceps biopsy, and a combination of both methods to differentiate ECC from benign biliary disease in the Trefle^®^ and POCS groups. The values for sensitivity, specificity, positive predictive value (PPV), negative predictive value (NPV), and accuracy of Trefle^®^-assisted tissue acquisition were 87.5%, 83.3%, 87.5%, 83.3%, and 85.7%, respectively; and for POCS-guided forceps biopsy, the values for all the above-mentioned parameters were 90.0%. There was no significant difference in the sensitivity, specificity, PPV, NPV, and accuracy of Trefle^®^-assisted tissue acquisition and POCS-guided forceps biopsy for all the patients examined. 

### 3.3. Adverse Events

Adverse events associated with both Trefle^®^-assisted tissue acquisition and POCS-guided forceps biopsy are shown in Table 5. In this study, adverse events following Trefle^®^-assisted tissue acquisition occurred in five patients (35.7%), with three patients developing acute pancreatitis (21.4%), and all were mild; and two patients developing infection (cholangitis, 14.3%). In Trefle^®^ group, all the cases of adverse events occurred in patients without EST. Adverse events following POCS-guided forceps biopsy occurred in five patients (25.0%), with three patients developing acute pancreatitis (15.0%), including one case of severe pancreatitis; and one patient developing infections (cholangitis, 5.0%). Severe hemorrhage related to EST occurred in one patient (5.0%). There was no significant difference in the occurrence of adverse events between the Trefle^®^ group and the POCS group. No perforations were observed, all cases were resolved with conservative treatment, and there was no procedure-related mortality.

## 4. Discussion

ERCP is a common method of tissue acquisition in patients with biliary strictures, using bile aspiration cytology, biliary brush cytology, and forceps biopsy. As reported in recent studies, the sensitivity of these endoscopic tissue acquisition methods for malignant biliary stricture is 41.6%, 45.0%, and 48.1%, respectively [4,5]. Navaneenthan et al. reported that a combination of biliary brush cytology and forceps biopsy only modestly increased the sensitivity to 59.4% [5]. Every tissue acquisition method is almost 100% specific [5]. Recently, the efficacy of endoscopic ultrasound-guided fine-needle aspiration (EUS-FNA) for tissue acquisition in malignant biliary stricture was reported [10,11,12]. Weilert at al. reported that EUS-FNA is superior to ERCP-guided tissue sampling in accurately diagnosing a suspected malignant biliary obstruction [10]. Sadeghi at al. also reported a low rate of adverse side effects (bleeding, 1.0%; biliary peritonitis, 0.3%) when using EUS-FNA for the diagnosis of malignant biliary strictures [11]. However, the possibility of needle track seeding using EUS-FNA in resectable cases remains unresolved [13,14]. Furthermore, the transpapillary procedure remains the standard procedure for the diagnosis and drainage of biliary strictures, especially in obstructive jaundice patients. Although ERCP tissue acquisition plays an important role in the diagnosis of indeterminate biliary stricture, the poor sensitivity of this method makes it inadequate for clinical use. To improve the sensitivity for ECC, multiple sampling strategies, such as immunohistochemistry testing, mutational analysis, digital image analysis, and fluorescence in situ hybridization have been used. However, the specificity for the diagnosis of ECC in these methods is also insufficient [15,16].

Recently, POCS has been an increasingly-used technique in diagnosing indeterminate biliary stricture. POCS allows optical viewing of the biliary system, as well as targeted biopsies under direct vision. Previous studies have suggested that the use of POCS can improve the diagnostic accuracy of indeterminate biliary strictures [6,17]. Furthermore, Nishikawa et al. reported that visual findings for POCS had higher sensitivity and accuracy in diagnosing indeterminate biliary lesions than those of cholangioscopy-guided forceps biopsy [18]. However, the sensitivity of POCS-guided forceps biopsy for malignant biliary stricture was still insufficient (60.1%) [9]. The working channel of POCS is narrow, so we could only use mini-forceps with a 1.0-mm diameter cup in POCS-guided forceps biopsy. A possible reason for the lack of sensitivity in the pathological diagnostic ability of POCS-guided forceps biopsy may be because the specimen obtained by POCS is relatively small. To improve the diagnostic performance for indeterminate biliary stricture, a working channel with a larger diameter for large capacity forceps might be needed [19]. When the cholangioscope was inserted into the bile duct, some procedure for biliary access, such as EST, is required. As a result, the sphincter of Oddi function was lost entirely. It is a disadvantage of POCS.

Trefle^®^ was designed to easily access biliary strictures and obtain both tissue specimens and cell samples for histological and cytological diagnosis. Although the sensitivity of Trefle^®^-assisted tissue acquisition for ECC was not satisfactory (64.1%), this device reduced the number of patients with insufficient/no samples [8]. The advantage of Trefle^®^-assisted tissue acquisition lies in its wire-guided system, which can be inserted into the bile duct and reaches the biliary stricture easily. This system allowed us to avoid unnecessary EST, which may help to prevent early adverse events, such as bleeding; and late adverse events, such as liver abscess [20,21].

In general, the indications of POCS-guided forceps biopsy were limited for the cases of difficulty in the diagnosis by ERCP-related tissue acquisition including transpapillary biliary forceps biopsy and brushing cytology. This main reason was the high cost of POCS. However, the sensitivity of ERCP-related tissue acquisition was unsatisfactory, we considered the initial POCS-guided forceps biopsy for indeterminate biliary stricture was acceptable if the sensitivity of POCS-guided forceps biopsy was significantly higher than that of Trefle^®^. It is important to avoid the excessive repetition of ERCP because of its high adverse event rate. 

In our study, the sensitivity of Trefle^®^-assisted tissue acquisition for ECC was shown to be similar to that of POCS-guided forceps biopsy. However, Trefle^®^-assisted tissue acquisition might be superior to POCS-guided forceps biopsy for the pathological diagnosis for indeterminate biliary stricture, Trefle^®^ preserves the sphincter of Oddi function. In this study, EST was performed for fewer patients in the Trefle^®^ group than in the POCS group. However, all the cases of adverse events, including pancreatitis following Trefle^®^-assisted tissue acquisition, occurred in patients without EST in our previous study [8]. EST might be negative factor associated with post-ERCP pancreatitis in the Trefle^®^ group. It is controversial whether or not EST are needed for the tissue acquisition procedure by Trefle^®^.

Superficial intraductal spread, in which the epithelium extends continuously from the primary lesion, is a feature of ECC [22]. Superficial intraductal spread in cholangiocarcinoma occurs in 14.6% of patients, according to previous research [23]. The presence of superficial intraductal spread is related to positive resection margins after surgery. Therefore, the preoperative identification of the exact perihilar and distal margins of resectable ECC is important. Hijioka et al. reported that the diagnostic yield of fluoroscopic mapping biopsy procedures (ERCP tissue acquisition) to accurately distinguish between benign and malignant foci was 89% [24]. ERCP-guided mapping biopsy for defining the longitudinal extension of ECC is an indispensable modality for patients with resectable ECC. Although the exact margins cannot be determined using Trefle^®^ tissue acquisition, POCS and POCS-guided mapping biopsy were also useful in the preoperative assessment of the longitudinal extension of ECC [25,26]. For these reasons, the initial pathological diagnosis for indeterminate biliary stricture should preferentially be made using Trefle^®^-assisted tissue acquisition, which has a decent sensitivity and enables surgeons to avoid unnecessary EST. If the biliary stricture was diagnosed as a resectable ECC, POCS-guided mapping biopsy might play an important role in the preoperative assessment of longitudinal extension of ECC after diagnosis of ECC. 

This study has some limitations. First, this was a retrospective, single-center study with a small number of cases. Second, patients who were diagnosed by a clinical follow-up were also included in this study. Therefore, in the study, it was uncertain that the benign biliary stricture cases were truly benign or not. A prospective randomized long-term study including a larger number of patients is required.

## 5. Conclusions

The diagnostic ability of Trefle^®^-assisted tissue acquisition for ECC is similar to that of POCS-guided tissue acquisition. Trefle^®^ may enable us to avoid unnecessary EST for the evaluation of extrahepatic biliary stricture.

## Figures and Tables

**Table 1:** Baseline characteristics of study patients.

Parameter	Biliary Disease (*n* = 34)
Age (range), years	71 (47–85)
Sex, male/female	19/15
Location of stricture (perihilar/distal)	12/22
Malignant	6/12
Benign	6/10
Length of stricture, mm	16.2 (1.2–46.0)
Acute cholangitis (presence/absence)	4/30
Total bilirubin, mg/dL	1.2 (0.3–25.4)
Tumor marker	
CEA, ng/mL	
Malignant	2.4 (1.2–8.3)
Benign	2.2(1.4–4.2)
CA19-9, U/mL	
Malignant	57.9 (0.8–11985) *
Benign	7.7 (1.6–71.6)
Malignant	18
cholangiocarcinoma	18
Benign	16
Benign biliary stricture	11
IgG4-associated sclerosing cholangitis	1
Primary sclerosing cholangitis	1
Intraductal papillary neoplasm of bile duct	1
Bile duct adenoma	1
Peribiliary cyst	1

Values are presented as number or median (range). Abbreviations: CEA, carcinoembryonic antigen; CA19-9, carbohydrate antigen 19-9; IgG4, immunoglobulin G subclass 4. * *p* < 0.001 compared with benign biliary disease.

**Table 2:** Characteristics of patients with biliary disease.

	Trefle^®^ (*n* = 14)	POCS (*n* = 20)	*p* Value
Age, years	70.5 (53–85)	71 (47–84)	0.779 *^1^
Sex, male/female	8/6	11/9	0.901 *^2^
Malignancy/Benign	8/6	10/10	0.681*^2^
Location (perihilar/distal)	3/11	9/11	0.293 *^2^
Length of stricture, mm	18.4 (8.8–42.0)	16.0 (1.2–46.0)	0.431 *^1^
Acute cholangitis (presence/absence)	1/13	3/17	0.874 *^2^
Total bilirubin, mg/dL	1.1 (0.6–25.4)	1.3 (0.3–14.8)	0.972 *^1^
Tumor marker (serum)			
CEA, ng/mL			
Malignant	2.3 (1.2–4.8)	2.5 (1.4–8.3)	0.307 *^3^
Benign	2.0 (1.4–4.2)	1.9 (1.6–3.1)	0.609 *^3^
CA19-9, U/mL			
Malignant	76.5 (0.8–11985.0)	52.5 (17.8–577.6)	0.271 *^3^
Benign	7.7 (7.1–13.6)	7.6 (1.6–71.6)	0.545 *^3^

Values are presented as number or median (range). Abbreviations: POCS, peroral cholangioscopy; CEA, carcinoembryonic antigen; CA19-9, carbohydrate antigen 19-9. *^1^ *p* value: Mann–Whitney U test. *^2^ *p* value: Chi-square test. *^3^ *p* value: Student’s t-test.

**Table 3:** Procedure of Trefle^®^ tissue acquisition and POCS-guided forceps biopsy for differentiating extrahepatic cholangiocarcinoma from benign biliary disease.

	Trefle^®^ (*n* = 14)	POCS (*n* = 20)	*p* Value
Procedure time, minutes	92.0 (57–129)	85.5 (34–170)	0.766 *^1^
EST or precut papillotomy (previous/with/without)	1/3/10	5/15/0	<0.001 *^2^
Number of biopsy, times	2 (1–6)	3 (2–9)	0.0498 *^1^

Values are presented as number or median (range). Abbreviations: POCS, peroral cholangioscopy; EST, endoscopic sphincterotomy. *^1^ *p* value: Mann–Whitney U test. *^2^ *p* value: Chi-square test.

**Table 4:** Diagnostic ability of Trefle^®^ tissue acquisition and POCS-guided biopsy for differentiating extrahepatic cholangiocarcinoma from benign biliary disease.

		Sensitivity, %	Specificity, %	PPV, %	NPV, %	Accuracy, %
Trefle^®^	BAC	87.5	100	100	85.7	92.9
		(7/8)	(6/6)	(7/7)	(6/7)	(13/14)
	TB	62.5	83.3	83.3	62.5	71.4
		(5/8)	(5/6)	(5/6)	(5/8)	(10/14)
	BAC + TB	87.5	83.3	87.5	83.3	85.7
		(7/8)	(5/6)	(7/8)	(5/6)	(12/14)
POCS	BAC	40.0	100	100	60.0	68.4
		(4/10)	(9/9)	(4/4)	(9/15)	(13/19)
	PB	90.0	90.0	90.0	90.0	90.0
		(9/10)	(9/10)	(9/10)	(9/10)	(18/20)
	BAC + PB	90.0	90.0	90.0	90.0	90.0
		(9/10)	(9/10)	(9/10)	(9/10)	(18/20)

Abbreviations: BAC, bile aspiration cytology; TB, Trefle^®^-assisted biopsy; PB, POCS-guided forceps biopsy; PPV, positive predictive value; NPV, negative predictive value. *p* value: Chi-square test.

**Table 5:** Adverse events with Trefle^®^-assisted tissue acquisition and POCS-guided forceps biopsy.

Adverse Event	Trefle^®^ (*n* = 14)	POCS (*n* = 20)	*p* Value
Pancreatitis	21.4 (3/14)	15.0 (3/20)	0.672
Bleeding	0	5.0 (1/20)	1.000
Infection	14.3 (2/14)	5.0 (1/20)	0.555
Perforation	0	0	NS
Cardiac	0	0	NS
Pulmonary	0	0	NS
Medication reaction	0	0	NS
Other	0	0	NS
Overall	35.7 (5/14)	25.0 (5/20)	0.704

Abbreviation: NS, not significant. *p* value: Fisher’s exact test.
